# Effect of Cross-Sex Hormonal Replacement on Antioxidant Enzymes in Rat Retroperitoneal Fat Adipocytes

**DOI:** 10.1155/2016/1527873

**Published:** 2016-08-18

**Authors:** Israel Pérez-Torres, Verónica Guarner-Lans, Alejandra Zúñiga-Muñoz, Rodrigo Velázquez Espejel, Alfredo Cabrera-Orefice, Salvador Uribe-Carvajal, Natalia Pavón

**Affiliations:** ^1^Department of Pathology, Instituto Nacional de Cardiología “Ignacio Chavez”, Juan Badiano 1, Sección XVI, Tlalpan, 14080 México City, Mexico; ^2^Department of Physiology, Instituto Nacional de Cardiología “Ignacio Chavez”, Juan Badiano 1, Sección XVI, Tlalpan, 14080 México City, Mexico; ^3^Department of Molecular Genetic, Instituto de Fisiología Celular, Universidad Nacional Autónoma de México, Coyoacán, 04510 México City, Mexico; ^4^Department of Pharmacology, Instituto Nacional de Cardiología “Ignacio Chavez”, Juan Badiano 1, Sección XVI, Tlalpan, 14080 México City, Mexico

## Abstract

We report the effect of cross-sex hormonal replacement on antioxidant enzymes from rat retroperitoneal fat adipocytes. Eight rats of each gender were assigned to each of the following groups: control groups were intact female or male (F and M, resp.). Experimental groups were ovariectomized F (OvxF), castrated M (CasM), OvxF plus testosterone (OvxF + T), and CasM plus estradiol (CasM + E_2_) groups. After sacrifice, retroperitoneal fat was dissected and processed for histology. Adipocytes were isolated and the following enzymatic activities were determined: Cu-Zn superoxide dismutase (SOD), catalase (CAT), glutathione peroxidase (GPx), glutathione-S-transferase (GST), and glutathione reductase (GR). Also, glutathione (GSH) and lipid peroxidation (LPO) were measured. In OvxF, retroperitoneal fat increased and adipocytes were enlarged, while in CasM rats a decrease in retroperitoneal fat and small adipocytes are observed. The cross-sex hormonal replacement in F rats was associated with larger adipocytes and a further decreased activity of Cu-Zn SOD, CAT, GPx, GST, GR, and GSH, in addition to an increase in LPO. CasM + E_2_ exhibited the opposite effects showing further activation antioxidant enzymes and decreases in LPO. In conclusion, E_2_ deficiency favors an increase in retroperitoneal fat and large adipocytes. Cross-sex hormonal replacement in F rats aggravates the condition by inhibiting antioxidant enzymes.

## 1. Introduction

Sexual steroid hormones are the basis of many physiological and pathophysiological processes [1]. Experimental and clinical studies suggest that sex hormones participate in maintenance and distribution of body fat mass. In women, there is usually an increase in adipocyte number after menopause, when estrogens decrease to levels similar to those in men [2]. Fat tissue in premenopausal women is located primarily in subcutaneous deposits; however, at menopause, visceral adiposity ensues [3]. In animal models (mice), obesity is associated with menopause, which is sensitive to estradiol (E_2_) therapy or ovariectomy, since it can be reversed through the administration of E_2_ [4].

By contrast, the role of testosterone (T) in obesity is not well established and it may depend on its circulating concentration. Previous reports have described that serum T concentrations below the baseline level in healthy young eugonadal men are associated with high intra-abdominal adipose tissue mass [5]. Low T concentrations are associated with loss of androgen receptors and a higher risk of type 2 diabetes [6]. Adipose tissue is no longer considered just a fat deposit, but it is, instead, one of the largest endocrine organs, producing a variety of bioactive molecules such as adipokines, cytokines, and hormones [1]. However, during obesity, visceral adipose tissue promotes synthesis of proinflammatory cytokines, which cause an increase in the level of reactive oxygen species (ROS) [7].

ROS can be generated in several intracellular sites, including peroxisomes, plasma membrane and endoplasmic reticulum (ER), and the cytoplasm. ROS can attack biomembranes, enzymes, proteins, and nucleic acids. Their reactivity can be neutralized by antioxidant systems, engaging in a delicate balance which determines the impact of ROS in cells [8]. The superoxide anion (O_2_^−^) is a ROS, which is detoxified by superoxide dismutase (SOD) to H_2_O_2_. Catalase (CAT) and glutathione peroxidase (GPx) further degrade this end product to water [8]. In addition, the glutathione family includes GPx, glutathione-S-transferase (GST), and glutathione reductase (GR). GPx uses the reduced glutathione (GSH) as an H^+^ donor to degrade peroxides during the reduction of H_2_O_2_ to molecular H_2_ and O_2_. GSH is oxidized to glutathione oxidized (GSSG) [9]. GR uses a donor proton from NADPH to rereduce GSSG to GSH [9, 10]. In addition, GSH, the major intracellular thiol compound, is ubiquitous tripeptide produced by most mammalian cells and it is the main mechanism of antioxidant defense against ROS and electrophiles [10]. Several studies have reported that premenopausal females have a higher antioxidant potential in comparison to males when considering the concentrations of all antioxidants enzymes including SOD, CAT, GPx, GST, and GSH [11]. Regretfully, this advantage is lost with the decline of ovarian hormones and might play an important role in menopause-associated obesity. The aim of this study was to determine if the cross-sex hormonal replacement modifies antioxidant enzyme activities in retroperitoneal fat adipocytes. It is important to fully understand the association of sex hormone therapy with antioxidant enzymes.

## 2. Materials and Methods

Studies were conducted according to the laboratory animal care standards of our institution and in compliance with ethical guidelines for animal research. Rats were housed in ad hoc plastic boxes under 12-hour light/darkness cycles and a room temperature from 18 to 25°C. They were fed with commercial rodent pellets (23% crude protein, 4.5% crude fat, 8% ashes, and 2.55 added minerals, PMI nutrition international Inc., Lab Diet 5008, Richmond, VA) ad libitum. After overnight fasting, the animals were sacrificed with a guillotine and their blood was collected in vacutainer tubes. Samples were centrifuged for 20 min at 936 ×g and 4°C in order to collect the serum in aliquots and store it at −30°C.

### 2.1. Animals

24 rats (3 weeks old) of each gender with weights of 48 ± 5 g and 58 ± 8 g for female and male rats, respectively, were used in the experimental protocol; 8 rats were randomly assigned to each of the following groups: control intact female or male groups (F and M, resp.), ovariectomized female (OvxF) and castrated male (CasM), and treated Ovx female plus T (OvxF + T) or CasM plus E_2_ (CasM + E_2_).

### 2.2. Ovariectomy and Castration

These procedures were performed at 3 weeks of age of the animals. Surgical ovariectomy was performed under anesthesia (pentobarbital sodium 63 mg/Kg of body weight) as follows: the abdominal and pelvic areas of the back were shaved, cleaned, and disinfected with iodine. A longitudinal incision of 1.5 cm was made, the skin was separated from the muscle, and a second incision of 0.5 cm was made in the muscle on both sides of the first to exteriorize the ovaries. The Fallopian tubes were ligated and cut below the ligature. After the extirpation, the incision was closed. Surgical castration was performed under anesthesia (pentobarbital sodium 63 mg/Kg of body weight) as follows: the area of the scrotum was shaved, cleaned, and disinfected with iodine. A longitudinal incision of 1 cm was made, the efferent ducts of the testicles were ligated, and the testicles were removed. After extirpation the incision was closed.

### 2.3. Hormonal Treatment

E_2_ valerate or T enanthate (Primogyn, Schering, México; 1 mg/Kg body weight) was injected intramuscularly every 5 days for 4 months.

### 2.4. Measurement of Serum Sex Hormones and Cytokines

Serum E_2_ and T were measured using the Diagnostic Products Corporation Kits (Los Angeles, CA) and determination of proinflammatory cytokines, IL-1, IL-6, and TNF-*α*, in adipocyte homogenates was done by ELISA kits obtained from PeproTech.

### 2.5. Isolation of Adipocytes

White adipocytes were isolated by collagenase digestion as described by Rodbell [12] with the modifications described by Guerra et al. [11]. The samples were frozen at −30°C. Total proteins were determined by Bradford [13].

### 2.6. Superoxide Dismutase Activity

75 *μ*g adipocyte homogenate was applied directly, without boiling, to a nondenaturing 10% polyacrylamide gel. The electrophoresis was carried out at 120 volts for 4 hours. Subsequently, the gel was incubated in a 2.45 mM nitro blue tetrazolium solution for 20 min; then the liquid was discarded and the gel was incubated in a 28 mM EDTA solution, containing 36 mM potassium phosphate (pH 7.8) and 0.028 mM riboflavin. After 10 min of incubation under dark conditions, the nitro blue tetrazolium stain for O_2_ was viewed by UV light exposure for another 10 min. The gels were analyzed by densitometry by the image analyzer SigmaScan Pro 5 [11].

### 2.7. Catalase Activity

75 *μ*g adipocyte homogenate was applied directly to a nondenaturing 10% polyacrylamide gel. The electrophoresis was carried out at 120 volts for 4 hours. Subsequently, the gel was washed with distilled water for 5 minutes; this procedure was repeated three times; then it was incubated with 0.03% H_2_O_2_ for 10 minutes. Then it was incubated with a mixture of 1% K_3_Fe(CN_6_) and 1% of FeCl_3_ 6H_2_O for 10 minutes in dark and then washed with distilled water to stop the reaction. The gels were analyzed by densitometry by the image analyzer SigmaScan Pro 5 [11].

### 2.8. Glutathione Peroxidase

For GPx activity, 100 *μ*L of adipocyte homogenate was suspended in 1.6 mL of 50 mM phosphate buffer (pH 7.3), to which 0.2 mM NADPH, 1 mM GSH, and 1 UI/mL glutathione reductase were added. The mixture was incubated for 3 minutes at 37°C; then 100 *μ*L of 0.25 mM H_2_O_2_ was added to start the reaction and absorbance was monitored for 10 min at 340 nm [11]. Activity is expressed in *μ*mol NADPH oxidized/min/mg protein.

### 2.9. Glutathione-S-Transferase

700 *μ*L phosphate buffer (0.1 M, pH 6.5) supplemented with 100 *μ*L GSH 0.1 mM and 100 *μ*L 1-chloro-2,4-dinitrobenzene (CDNB) 0.1 mM was added to 100 *μ*g of adipocyte homogenate. The sample was incubated and monitored at 340 nm for 10 min at 37°C. Values of GST activity were expressed in U/min/mg. A unit of activity of GST is expressed in *μ*mol of GS-DNB conjugate formed/min/mg protein [14].

### 2.10. Glutathione Reductase

To evaluate GR activity, 700 *μ*L of phosphate buffer 0.2 mM, plus 0.5 mM of EDTA pH 7.3, 100 *μ*L of NADPH 0.1 mM, and 100 *μ*L of GSSG 1 mM, was added to 100 *μ*g of adipocyte homogenate. It was then incubated and monitored for 10 min at 37°C and the absorbance was read at 340 nm. GR activity is expressed in U/min/mg protein [15].

### 2.11. GSH Concentration

To determine GSH concentration, 800 *μ*L of phosphate buffer 50 mM, pH 7.3, plus 100 *μ*L of Ellman reactive (5,5′ dithiobis 2-nitrobenzoic) 1 M, was added to 100 *μ*g of adipocyte homogenate previously deproteinized with 20% trichloroacetic acid (vol/vol) and centrifuged to 5000 rpm for 5 minutes. The mixture was incubated at room temperature for 5 minutes and absorbance was read at 412 nm. The calibration curve was made with GSH at 5 to 25 *μ*mol/mg protein [16].

### 2.12. Lipid Peroxidation

50 *μ*L CH_3_-OH with 4% BHT plus phosphate buffer pH 7.4 was added to 100 *μ*g of adipocyte homogenate. The mixture was shaken vigorously in vortex for 5 seconds and then incubated in a water bath at 37°C for 30 min. 1.5 mL of 0.8 M thiobarbituric acid was added and the sample was incubated in a water bath at boiling temperature for 1 hour. After this time and to stop the reaction, the samples were placed on ice; 1 mL 5% KCl was added to each sample as well as 4 mL n-butanol; they were shaken in vortex for 30 seconds and centrifuged at 4000 rpm at room temperature for 2 min. Then the n-butanol phase was extracted and the absorbance was measured at 532 nm. The calibration curve was obtained using tetraethoxypropane as standard [11].

### 2.13. Retroperitoneal Fat Histology

For histology, 2 mm of retroperitoneal fat was washed in 0.9% NaCl for 30 seconds. The solution was then decanted and phosphate buffer with 10% formalin was added for 24 hours. The histological sections were processed according to conventional histological procedures and stained with Masson [17]. Histological sections were analyzed using a light microscope Carl Zeiss (63300 model) equipped with a Tucsen (9 megapixels) digital camera with software TSview 7.1, at a 40x magnification. The photomicrographs were analyzed by densitometry using SigmaScan Pro 5 image analysis software. The density values are expressed as pixel units.

### 2.14. Statistical Analysis

Statistical analysis and graphics were performed with the SigmaPlot 11 program, Jendel Corporation, 1986–2010. The data are presented as the mean ± SEM. Statistical significance was determined by one-way ANOVA test, followed by Tukey's post hoc test. Differences were considered as statistically significant at *p* < 0.05.

## 3. Results and Discussion

Postmenopausal women and men are more obese than premenopausal women [18] and E_2_ protects against the development of insulin resistance, diabetes, and cardiovascular diseases, while T has the opposite effects [19]. Retroperitoneal fat accumulates more than subcutaneous adipose tissue. It is metabolically active and is liberated to the portal circulation substances such as inflammatory cytokines and free fatty acids, which lead to insulin resistance, hypertension, and cardiometabolic risk [20]. Symptoms of these diseases appear in women at older ages than in men and the risk in women is doubled if they have undergone hysterectomy plus oophorectomy. This increase does not occur when ovaries are preserved [4]. The absence of female sex hormones in menopause often leads to changes in body weight [21] and increased retroperitoneal fat deposits [22]. Our experimental rat model, characterized by mild obesity is useful to study the influence of sex hormone deficiency on adiposity and for the study of sex hormonal replacements [22]. Furthermore, this is considered a very useful model for postmenopausal and hypoandrogenic conditions. Thus, we decided to evaluate the influence of cross-sex hormonal replacement on the antioxidant enzymes contained in rat retroperitoneal fat adipocytes.

### 3.1. Sex Hormones

There were significant differences in E_2_ concentration in serum in intact F group versus OvxF and OvxF + T groups (*p* = 0.05 and *p* = 0.01, resp.) (Table 1). E_2_ concentration in intact M group decreased in comparison with the CasM and CasM + E_2_ groups (*p* = 0.01, Table 1). T concentration in serum increased significantly in OvxF + T group versus intact F rats (*p* = 0.05). In CasM and CasM + E_2_ rats, there was a significant decrease in T reaching nondetectable levels (*p* = 0.001). Results suggest that castration in males significantly increased the levels of serum E_2_, possibly due to extragonadal aromatization in adipose tissue [23]. Aromatization of androgens in adipose tissue has been associated with central gynecoid fat distribution [24].

### 3.2. Retroperitoneal Fat

Figure 2(c) shows that the amount of retroperitoneal fat was similar in intact F and OvxF groups; however, in OvxF + T group, there was a significant increase (*p* < 0.001). Likewise, there were no significant changes in intact M and CasM groups but treatment with E_2_ increased retroperitoneal fat (*p* = 0.001). These results suggest that the elimination of E_2_ plus T treatment led to increased obesity, associated with larger adipocytes size in female rats. In M rats, castration plus treatment with E_2_ led to increased obesity but associated with small adipocytes size. Sex hormone deficiency or replacement may elicit a disruption in gene regulation which results in cell proliferation and changes in size of adipocytes. E_2_ can prevent lipogenesis by decreasing the expression of SREBP-1c in adipose tissue and by reducing LXR-*α* expression, which is a positive regulator of SREBP-1c and would promote expression of lipogenic genes such as FAS and ACC-1 [25]. E_2_ also suppresses lipoprotein lipase transcription possibly due to an estrogen response element located in the promoter region of this gene [26].

### 3.3. Histology of the Retroperitoneal Fat Tissue

**Figure 1:**
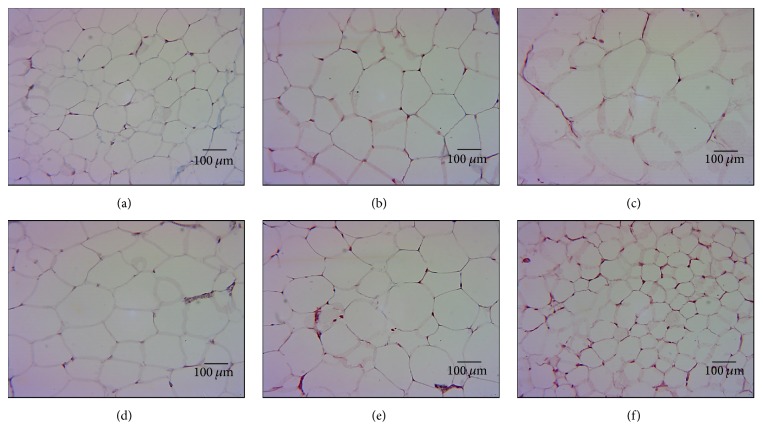
(a, b, c, d, e, and f) Representative photomicrographs of visceral white tissue from the experimental groups that show adipocyte size. 10 fields per sample were analyzed. (a) = F, (b) = OvxF, (c) = OvxF + T, (d) = M, (e) = CasM, and (f) = CasM + E_2_. Values are the mean ± SE (*n* = 8). The tissue was processed according to conventional histological procedures and histological sections were made and stained by Masson technique at 40x.

**Figure 2:**
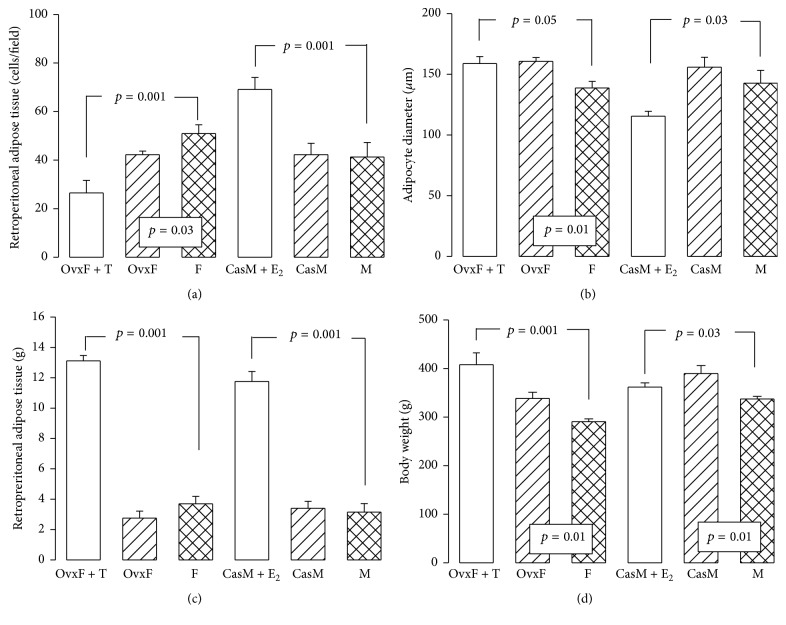
(a, b, c, and d) Representative histograms of retroperitoneal adipose cell by field (a), adipocyte diameter (b), retroperitoneal adipose tissue (c), and body Weight (d). F, Female; OvxF, ovariectomized female; OvxF + T, ovariectomized female plus testosterone; M, male; CasM, castrated male; CasM + E_2_, castrated male plus estradiol. Data are means ± SE; *n* = 8 rats by experimental group.

Micrographs of retroperitoneal adipose tissue show that, in F, adipocytes contained an empty cytoplasm limited by the cell membrane and the nucleus was small and near the periphery. An average of 50 ± 3 cells was found per field and the adipocyte diameters were 138 ± 5 *μ*m (Figures 1(a), 2(a), and 2(b), resp.). In OvxF group, retroperitoneal adipose tissue showed an empty cytoplasm limited by the cell membrane and the nucleus was small and in the periphery (Figure 1(b)); the average number of cells per field was lower, 42 ± 1, and the adipocyte diameters increased to 160 ± 3 *μ*m (Figures 2(a) and 2(b)), showing a significant difference in comparison to intact F group (*p* = 0.03 and *p* = 0.01, resp.). In OvxF + T group, the retroperitoneal adipose tissue contains irregular and large adipocytes with an empty cytoplasm surrounded by a thick cell membrane; the nucleus was also small and peripheral. Cell size was of 26 ± 5 cells per field and the adipocyte diameters were 158 ± 5 *μ*m with a significant difference as compared to the F group (Figures 1(c), 2(a), and 2(b), resp.). The M group (Figure 1(d)) exhibited large adipocytes with an empty cytoplasm limited by the cell membrane and a small peripheral nucleus. An average of 42 ± 6 adipocytes per field with a diameter of 142 ± 10 *μ*m (Figures 2(a) and 2(b), resp.) was found. CasM (Figure 1(e)) had irregular adipocytes and an average of 42 ± 4 cells per field and diameters of 155 ± 8 *μ*m, similar to those found in the M group. CasM + E_2_ (Figure 1(f)) exhibited irregular, small adipocytes and an average of 115 ± 4 cells per field with diameters of 69 ± 5 *μ*m with significant changes when compared to the intact M group (Figures 2(a) and 2(b), *p* = 0.001 and *p* = 0.03, resp.). These changes in adipocyte size in the CasM + E_2_ group can be associated with the action of E_2_ directly inhibiting the deposition of adipose tissue and by decreasing lipogenesis. This process may be inhibited by a decrease in mRNA, activity, and expression of lipoprotein lipase. This enzyme regulates the storage of triglycerides in the adipocytes [11]. In the female mice model subjected to ovariectomy, E_2_ replacement can prevent adipocyte hypertrophy [27]. Furthermore, the hormone replacement therapy may decrease the postmenopausal growth of fat mass by about 60%. This action is related to a dual effect of E_2_ or to its direct effect on tissues involving lipid metabolism [28]. Attenuation in retroperitoneal fat mass has been attributed to expression of *α* and *β* estrogenic receptors in retroperitoneal fat. However, *α* receptor expression is predominant, decreasing during central obesity [3]. E_2_ may stimulate the expression of *α* receptor as described in ER*α*KO and ARKO mice, where a decrease in systemic E_2_ levels can cause a significant increase in abdominal adiposity [4]. The changes in adipocyte size in OvxF and OvxF + T groups show that T alters adipocyte lipid metabolism and differentiation. The androgen receptor in adipocytes may be responsible for adiposity in the male [29]; this receptor mediates transcriptional activation of downstream genes that regulate lipid metabolism [19]. Chronic T treatment induces selective insulin resistance in subcutaneous adipocytes of women and is associated with an increase in the androgen receptor [19]. In addition, in prenatal female monkeys exposed to diverse concentrations of T, there was an increase in visceral adiposity associated with the presence of the androgen receptor [30]. In orchidectomized mice, dihydrotestosterone treatment results in androgen receptor-mediated obesity [31]. Also, women with polycystic ovary syndrome are characterized by an increase in visceral adipose tissue and an increase in the androgen receptor, suggesting that, in women, high T might increase both fat mass and the androgen receptor expression [31]. The increased retroperitoneal fat was associated with small or large adipocytes in CasM + E_2_ and OvxF + T groups, respectively. Small adipocytes are more strongly associated with insulin sensitivity than larger adipocytes. Moreover, the up or down expression of genes that participate in expression of enzymes of lipogenesis is correlated with adipocyte size [32]. Large adipocytes linked to T and E_2_ have a prominent effect on genes regulating adipocyte morphology [32]. An increase in larger adipocytes has been described in Ovx female C57BL/6 mice in comparison with intact female and Ovx female supplemented with E_2_. These were also associated with higher levels of inflammatory markers such as CD68 and TNF-*α* and with increases in oxidative stress [33]. Other authors have described that Ovx mice and postmenopausal women have large adipocytes in spite of having a high lipolytic activity and this can potentially lead to a hypoxic environment. This hypoxic setting can increase adipocyte oxidative stress, producing ROS, which can lead to damage of genes encoding for antioxidant enzymes [33].

### 3.4. Body Weight

The weight of the retroperitoneal fat and size of adipocytes can be associated with body weight in rats.  Figure 2(d) shows that body weight in F group was significantly smaller (*p* = 0.01 and *p* = 0.001, resp.) than in OvxF and OvxF + T groups. In the M group, a similar tendency was observed in comparison to the CasM and CasM + E_2_ groups (*p* = 0.01 and *p* = 0.03, resp.). A body weight increase in several conditions can be associated with E_2_ deficiency. Ovariectomy, polycystic ovary syndrome, or the lack of a functional aromatase gene can be corrected by E_2_ treatment [25]. On the other hand, adipose tissue is no longer considered solely as a fat deposit. It is now considered as one of the largest endocrine organs producing a variety of bioactive factors such as cytokines (e.g., tumor necrosis factor-*α* (TNF-*α*) and interleukin-6 (IL-6)). These cytokines act in either paracrine or endocrine manners to regulate lipid metabolism and inflammation [7]. Several research groups have demonstrated that adipose tissue of obese animals and humans produces large amounts of inflammatory mediators and contains more inflammatory cells than lean controls [34]. Excess retroperitoneal fat is probably associated with increased oxidative stress in the adipocyte [35]. Under excess ROS, the endoplasmic reticulum of adipocytes accumulates misfolded or unfolded proteins that increase oxidative stress in adipose tissue [35]. Excess retroperitoneal adipose tissue is also a source of inflammatory cytokines such as IL-1, IL-6, and TNF-*α* and thus obesity is considered as a chronic inflammatory state. These cytokines are a potent stimulus for a positive feedback in the production of ROS [36].

### 3.5. Cytokines

Table 1 shows variations in cytokine levels in the different experimental groups. IL-1 concentrations in serum were similar in the F and M rats. IL-6 concentration in serum was significantly decreased in CasM + E_2_ group as compared to M group (*p* < 0.05). TNF-*α* concentration in the serum did not vary in any of the F groups but showed significant decreases in the CasM and CasM + E_2_ groups when compared to M group (*p* < 0.001).

Postmenopausal women have a higher incidence of abdominal adiposity, associated with an increase in systemic levels of inflammatory cytokines and ROS. This suggests that E_2_ may modulate body fat distribution and systemic inflammation [33]. Our results show that the TNF-*α* concentration tends to increase in OvxF and OvxF + T groups without showing a statistical difference; however, in CasM and CasM + E_2_ groups, it significantly decreased. These results are in agreement with previously described results, where E_2_ protected adipocytes against inflammation and oxidative stress in the female mice in comparison to intact males and ovariectomized females subjected to a high fat diet for 10 weeks [33]. IL-1, IL-6, and TNF-*α* are synthesized by macrophages that infiltrate organs undergoing chronic inflammation. E_2_ has potent anti-inflammatory properties and suppresses the expression of TNF-*α* [32, 33]. This suggests that the loss of E_2_ by ovariectomy favors a proinflammatory state in adipocytes of OvxF and OvxF + T groups, but male castration combined with E_2_ treatment slows this condition. Our data reinforce the notion that E_2_ prevents both body adiposity and systemic inflammation [32]. Moreover, ROS increased the adipocyte expression of MCP-1, a chemoattractant for monocytes and macrophages. Several subproducts of lipid peroxidation induced by ROS, such as 4-hydroxynonenal and malondialdehyde, are potent chemoattractants [36]. They increased ROS production and MCP-1 secretion from accumulated fat and caused infiltration of macrophages and inflammation in adipose tissue during obesity [37]. In this sense, in cultured adipocytes, H_2_O_2_ production increased in parallel with IL-6, while antioxidant enzymes decreased [37]. ROS can be generated in several sites including cytosol, peroxisomes, endoplasmic reticulum, and the plasma membrane [38]. ROS can attack biomembranes, enzymes, proteins, and nucleic acids. These oxidative effects can be neutralized by antioxidant systems [38]. The increased ROS levels after menopause are associated with the loss of endogenous estrogen synthesis and antioxidant enzymes. The first line of defense against O_2_^−^ is the cytosolic Cu-Zn SOD, which detoxifies O_2_^−^ to produce H_2_O_2_ [39].

### 3.6. Antioxidant Enzymes

#### 3.6.1. SOD Activity

**Figure 3:**
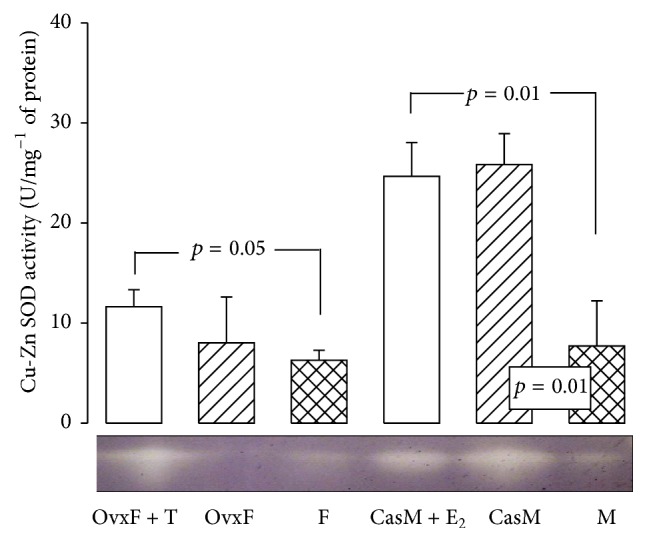
Effect of the cross-sex hormonal replacement on Cu-Zn SOD activity in adipocyte homogenate. F, female; OvxF, ovariectomized female; OvxF + T, ovariectomized female plus testosterone; M, male; CasM, castrated male; CasM + E_2_, castrated male plus estradiol; Cu-Zn SOD, superoxide dismutase copper-zinc. Native gel electrophoresis with 10% polyacrylamide. The lower panel is a native gel representative of the Cu-Zn SOD activity. The whole scanning shown represents the activity of the enzyme. Data are means ± SE; *n* = 8 rats in each group.

The Cu-Zn SOD activity in adipocyte homogenates did not show differences between F rats and OvxF rats, but treatment with T significantly increased its activity (*p* = 0.05).  Figure 3 also shows that the Cu-Zn SOD activity increased in CasM and CasM + E_2_ in comparison with M (*p* = 0.01). These results suggest that E_2_ and T modify SOD activity in adipocytes. Physiological concentrations of E_2_ and E_2_ receptors can decrease H_2_O_2_ through the MAPK and NF*κ*B pathways that modify the expression of antioxidant enzymes such as SOD and GPx [40]. E_2_ modulates the nuclear transcription factor, Nrf2, which controls the expression and induction of genes that encode for SOD and GST [41]. In liver fractions from 24-month-old menopausal female rats, treatment with E_2_ normalized the SOD activity [42]. Once produced, detoxification of cytoplasmic H_2_O_2_ occurs mainly through the CAT and GPx pathways.

#### 3.6.2. Catalase Activity

**Figure 4:**
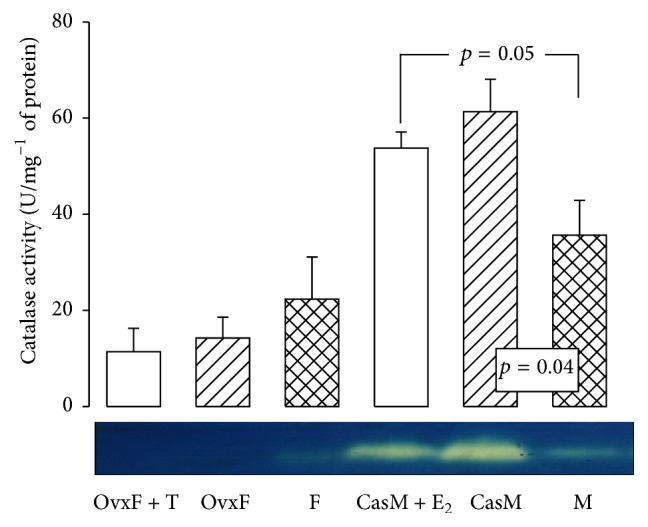
Effect of the cross-sex hormonal replacement on CAT activity in adipocyte homogenate. F, female; OvxF, ovariectomized female; OvxF + T, ovariectomized female plus testosterone; M, male; CasM, castrated male; CasM + E_2_, castrated male plus estradiol. Native gel electrophoresis with 10% polyacrylamide. CAT: catalase. The lower panel is a native gel representative of the CAT activity. The whole scanning shown represents the activity of the enzyme. Data are means ± SE; *n* = 8 rats in each group.

There were no differences in the activity of CAT in the adipocyte homogenate between intact F, OvxF, and OvxF + T groups, even if there was a slight tendency to decrease. In contrast, a significant increase was observed in the activity of CAT in CasM group as compared to M group (*p* = 0.04, Figure 4). Conversely, a significant increase in CAT in CasM + E_2_ group was observed. Females have been found to have less oxidative stress in the brain and an increased activity of CAT than males [43, 44]. However, in ovariectomized rats, a significant decrease in CAT activity was reported [45]. The ability of CAT to remove H_2_O_2_ depends on ovarian hormones that decline at menopause [44, 45]. In the kidney of Ovx female rats with metabolic syndrome, a decrease in CAT and Cu-Zn SOD activity has been reported but treatment with E_2_ favors an increase in their activities [46]. In adipocytes of ovariectomized female rats with metabolic syndrome, E_2_ increases the activities of SOD, CAT, and GPx [11]. E_2_ effect on CAT may occur through Nrf2 which mediates the basal expression of the genes of antioxidant enzymes involved in the oxidative stress response [11]. CAT and isocitrate dehydrogenase 1 are upregulated 3 weeks after E_2_ exposure; that is, E_2_ protects against oxidative stress [47]. GPx is another enzyme that detoxifies H_2_O_2_.

### 3.7. Glutathione Peroxidase

**Figure 5:**
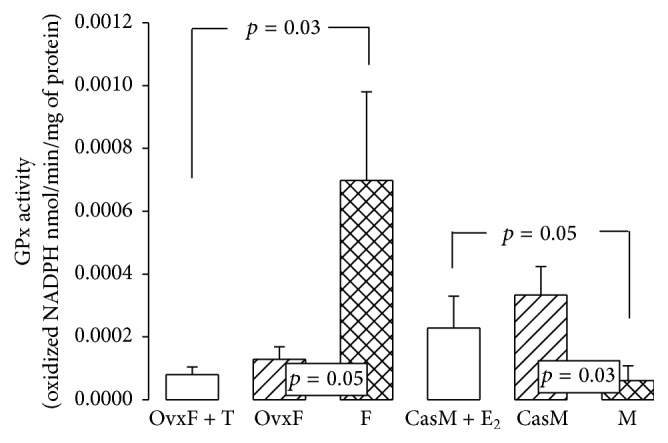
Effect of the cross-sex hormonal replacement on glutathione peroxidase activity in the adipocyte homogenate. Data are means ± SE; *n* = 8 rats in each group.

In F rats, GPx was increased in comparison to OvxF and OvxF + T groups (*p* = 0.05 and *p* = 0.03, resp.). In M group, GPx activity was significantly less than that in the CasM and CasM + E_2_ groups (*p* = 0.03 and *p* = 0.05, resp., Figure 5). The retroperitoneal adipose tissue from Ovx + E_2_ group exhibited an increase in GPx activity that was associated with adipocyte size [48]. GPx activity in rat livers from intact females is higher by 60% compared to that in OvxF group [49]. In murine skeletal muscle and adipose tissue, the genes that encode for GPx are sensitive to E_2_ and this effect is mediated through *α* receptors [50]. In addition, GPx activity was significantly higher in premenopausal women than in women after menopause [51]. In liver, GPx activity is significantly higher in females than in males, which is explained by the high GPx mRNA and selenium concentrations observed in females [44]. The ability of E_2_ to regulate GPx transcription may contribute to increased expression of GPx in females [49]. GPx transcription may be regulated directly or indirectly by Nrf2, as it enhances the expression of its gene [52]. Furthermore, the decrease in expression of GPx in adipocytes from obese mice may be due to chronic local inflammation with increased macrophage infiltration [53].

### 3.8. Glutathione-S-Transferase

**Figure 6:**
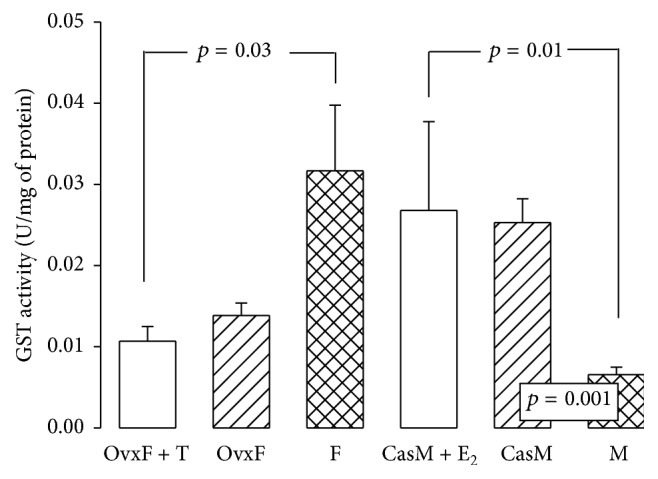
Glutathione-S-transferase activity in experimental groups. F, female; OvxF, ovariectomized female; OvxF + T, ovariectomized female plus testosterone; M, male; CasM, castrated male; CasM + E_2_, castrated male plus estradiol. The data are presented as mean ± SE; *n* = 8 rats in each group.

Other enzymes such as GST participate in ROS detoxification to prevent effects such as lipid peroxidation (LPO). This enzyme conjugates GSH to electrophilic agents, forming a bond and detoxifying them [54], and GST exhibits a wide intracellular distribution, being localized in mitochondria and cytosol and it may be bound to the cell membrane [55]. The results show that there were no statistically significant changes in GST activity from F group to OvxF group, but in Ovx + T group, GST activity decreased (*p* = 0.03). In CasM and CasM + E_2_ groups, there was a significant increase in GST activity as compared to M group (*p* = 0.001 and *p* = 0.01, resp., Figure 6). GSTA4 expression is selectively downregulated in the adipose tissue from obese insulin-resistant C57BL/6 J mice and in humans with obesity-linked insulin resistance. The mitochondrial function in adipocytes of lean or obese GSTA4-null mice was significantly compromised compared with wild-type controls and this was accompanied by an increase in O_2_^−^ production [56]. GST is intimately involved in the biosynthesis of T and progesterone [57]; however, the effect of the concentration of these hormones on their activity is unknown. GST Along with other antioxidant enzymes, such as Se-dependent GPx, GST protects the cell against oxidative damage [57]. Our results suggest that E_2_ increases GST activity in adipocytes, while T has the opposite effect. Effects may be mediated by Nrf2. This is supported by the fact that levels of mRNA encoding for GSTA1, GSTA2, GSTM1, and GSTM3 in the livers of Nrf2−/− mice fed on a normal diet have 20% less GST isoforms than Nrf2+/+ mice [58]. In mice, GST is regulated by Nrf2 [57] and this is associated with increased 4-hydroxynonenal, reduced antioxidant capacity, apoptosis, and low GSH [56].

In the detoxification of ROS, both GPx and GST used GSH. This is the major cellular thiol compound. It is ubiquitous tripeptide produced by most mammalian cells and it is the main antioxidant defense against ROS and electrophiles. Upon reaction with ROS or electrophiles, GSH becomes oxidized to GSSG, which can be reduced to GSH by GR [14]. The uncontrolled generation of GSSG during oxidative stress can lead to mitochondrial dysfunction by glutathionylation of proteins [59].

### 3.9. Glutathione Reductase Activity

**Figure 7:**
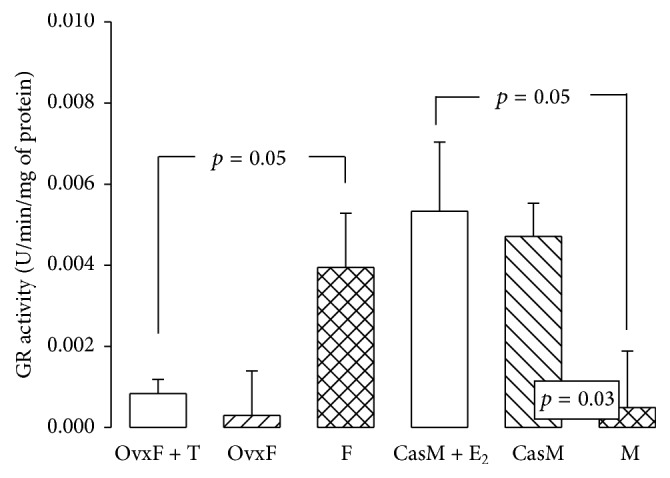
Effect of the cross-sex hormonal replacement on glutathione reductase activity in the adipocyte homogenate. Data are means ± SE; *n* = 8 rats in each group.

H_2_O_2_ is rapidly reduced to water mostly by GPx, which uses the reducing equivalents from its substrate GSH. In this enzymatic reaction, GSH becomes oxidized to GSSG, which is recycled back to GSH by the NADPH-dependent GR [60]. GR activity in the OvxF + T group decreased as compared to F group (*p* = 0.05). In the M group, the activity of GR was significantly less than that in CasM and CasM + E_2_ groups (*p* = 0.03 and *p* = 0.05, resp., Figure 7). The activity of GR is important to control the level of GSSG in the cell, since the uncontrolled generation of GSSG during oxidative stress, thus limiting the activity of GSH-dependent enzymes such as GPx and GST. Our results suggest that E_2_ and T can control GR activity in adipocytes. Furthermore, the accelerated loss of GSH that occurs under oxidative stress can be eventually restored by the modulation which E_2_ exerts on the GR activity recycling GSH in adipocytes E_2_ [10].

### 3.10. Glutathione

**Figure 8:**
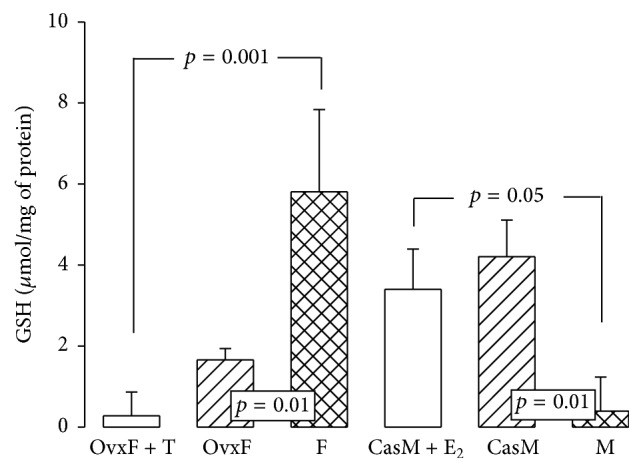
Reduced GSH concentrations in experimental groups. The data are presented as mean ± SE; *n* = 8 rats in each group.

Detoxification of H_2_O_2_ in mitochondria, cytosol, and plasma membrane occurs mainly through GSH, which is very important for redox balance in the cell [61]. GSH decreased in OvxF and OvxF + T groups versus F group (*p* = 0.01 and *p* = 0.001, resp.). In the M group, GSH was significantly lower than that in CasM and CasM + E_2_ groups (*p* = 0.01 and *p* = 0.05, resp., Figure 8). This suggests that E_2_ can act synergistically with GSH to protect cells from oxidative stress [62]. The antioxidant activity of E_2_ resides in the hydroxyl group at the C-3 position of the phenolic ring [62], which inhibits the oxidation cascades donating hydrogen atoms to lipid peroxyradicals. This interrupts the peroxidation chain reactions in membrane lipids [63]. In contrast, T lacks the C-3 position of the phenolic ring so it cannot act as an antioxidant molecule. Furthermore, the synergistic effect of E_2_ and GSH may be due to Nrf2, which is the main transcription factor regulating antioxidant response elements including *γ*-glutamylcysteine ligase which participates in GSH synthesis. The decrease in GSH may adversely affect cellular thiol redox balance contributing to oxidative stress [64]. GSH concentration depends on the equilibrium between its consumption and its biosynthesis. In postmenopausal women, a decrease in GSH was associated with its oxidation by increased ROS, and it counteracts the elevated levels of oxidative stress to inhibit membrane LPO [65].

### 3.11. Lipid Peroxidation

**Figure 9:**
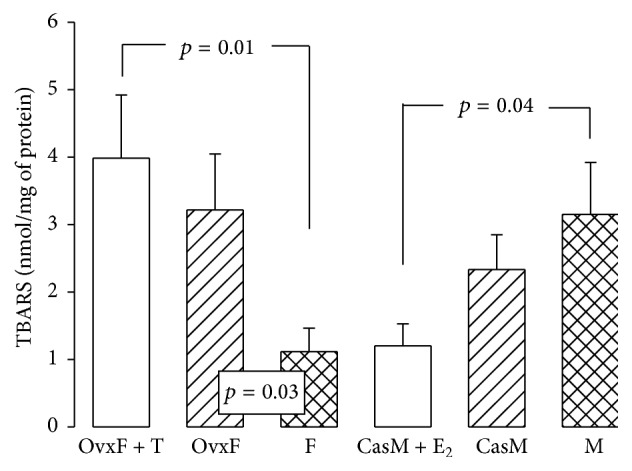
Lipid peroxidation was measured in adipocyte homogenate of the experimental groups. F, female; OvxF, ovariectomized female; OvxF + T, ovariectomized female plus testosterone; M, male; CasM, castrated male; CasM + E_2_, castrated male plus estradiol. Values are means ± SE.

LPO is a marker of damage by free radicals to the fatty acids in the phospholipids of the cellular membranes. Intracellular fat accumulation can disrupt mitochondrial function causing a buildup and subsequent leak of electrons from the electron transport chain that contributes to oxidative stress and LPO [9]. H_2_O_2_ induces LPO in adipocytes, which is attenuated by pretreatment with 10 nM E_2_ [66]. The LPO index in adipocyte homogenates increased significantly in OvxF and OvxF + T groups as compared to F group (*p* = 0.03 and *p* = 0.01, resp.). No significant changes were observed in the CasM group in comparison to M group. However, LPO decreased in CasM + E_2_ group as compared to M group (Figure 9). These results in the LPO index are the result of the effect of the cross-sex hormonal treatments on the antioxidant enzymes. In a murine model subjected to high fat diet, male mice and Ovx females had a significant increase in *γ*H2AX, a biomarker for oxidative stress, in the core of adipocytes compared to intact and Ovx females with E_2_ replacement. This was associated with an increase in LPO [33]. A product of LPO, 4-hydroxynonenal, was also significantly increased in postmenopausal women in comparison to premenopausal women suggesting that E_2_ protects against LPO. A possible explanation is that the key structure of the phenolic ring of E_2_ confers it with antioxidant properties [67]. Antioxidant actions of E_2_ on cell membranes are independent of the estrogen receptor. Thus the phenolic ring structure probably plays an important role [62]. E_2_ can also suppress LPO due to its similarity to vitamin E, while androgens have a prooxidant effect [68].

## 4. Conclusion

The removal of E_2_ by ovariectomy favors an increase in retroperitoneal fat, which is characterized by large adipocytes. In female rats, cross-sex hormone replacement aggravates this condition by altering antioxidant enzymes. In male rats, castration tends to decrease retroperitoneal fat accumulation which is characterized by small adipocytes. This decrease is further accentuated by the cross-sex hormone replacement, which promotes the activity of the antioxidant enzymes and decreases LPO.

## Figures and Tables

**Table 1:** General characteristics in experimental group.

Variables	F	OvxF	OvxF + T	M	CasM	CasM + E_2_
E_2_ (pg/mL)	102 ± 2	48 ± 5^*∗*^	11 ± 4^*∗∗*^	18 ± 3.4	54 ± 3^*∗∗*^	56 ± 2^*∗∗*^
T (ng/mL)	1 ± 0.1	1 ± 0.2	3 ± 0.3^*∗*^	8 ± 0.1	1 ± 0.1^†^	**<0.001** ^†^
IL-1 (ng/mL)	8 ± 3	10 ± 0	12 ± 4	9 ± 3	7 ± 1	6 ± 3
IL-6 (ng/mL)	1 ± 0.1	2 ± 0.0	1 ± 0.2	2 ± 0.4	2 ± 0.6	1 ± 0.3^*∗*^
TNF-*α* (ng/mL)	6 ± 1	9 ± 0.2	8 ± 3	31 ± 6	10 ± 4^†^	9 ± 3^†^

Data show the mean ± SEM; *n* = 8. F, female; OvxF, ovariectomized female; OvxF + T, ovariectomized female plus testosterone; M, male; CasM, castrated male; CasM + E_2_, castrated male plus estradiol. ^*∗*^ *p* < 0.05;  ^*∗∗*^ *p* ≤ 0.01: C versus MS; ^†^ *p* < 0.001.

## References

[1] Baños G., Guarner V., Pérez-Torres I. (2011). Sex steroid hormones, cardiovascular diseases and the metabolic syndrome. *Cardiovascular and Hematological Agents in Medicinal Chemistry*.

[2] Cooke P. S., Naaz A. (2004). Role of estrogens in adipocyte development and function. *Experimental Biology and Medicine*.

[3] Campbell S. E., Mehan K. A., Tunstall R. J., Febbraio M. A., Cameron-Smith D. (2003). 17*β*-Estradiol upregulates the expression of peroxisome proliferator-activated receptor *α* and lipid oxidative genes in skeletal muscle. *Journal of Molecular Endocrinology*.

[4] Heine P. A., Taylor J. A., Iwamoto G. A., Lubahn D. B., Cooke P. S. (2000). Increased adipose tissue in male and female estrogen receptor-*α* knockout mice. *Proceedings of the National Academy of Sciences of the United States of America*.

[5] Woodhouse L. J., Gupta N., Bhasin M. (2004). Dose-dependent effects of testosterone on regional adipose tissue distribution in healthy young men. *Journal of Clinical Endocrinology and Metabolism*.

[6] Tschöp M., Heiman M. L. (2001). Rodent obesity models: an overview. *Experimental and Clinical Endocrinology & Diabetes*.

[7] Zhou J., Qin G. (2012). Adipocyte dysfunction and hypertension. *American Journal of Cardiovascular Disease*.

[8] Abbas A. M., Elsamanoudy A. Z. (2011). Effects of 17*β*-estradiol and antioxidant administration on oxidative stress and insulin resistance in ovariectomized rats. *Canadian Journal of Physiology and Pharmacology*.

[9] Agarwal A., Aponte-Mellado A., Premkumar B. J., Shaman A., Gupta S. (2012). The effects of oxidative stress on female reproduction: a review. *Reproductive Biology and Endocrinology*.

[10] Ribas V., García-Ruiz C., Fernández-Checa J. C. (2014). Glutathione and mitochondria. *Frontiers in Pharmacology*.

[11] Guerra R. C., Zuñiga-Muñoz A., Guarner Lans V., Díaz-Díaz E., Tena Betancourt C. A., Pérez-Torres I. (2014). Modulation of the activities of catalase, Cu-Zn, Mn superoxide dismutase, and glutathione peroxidase in adipocyte from ovariectomised female rats with metabolic syndrome. *International Journal of Endocrinology*.

[12] Rodbell M. (1964). Metabolism of isolated fat cells. I. Effects of hormones on glucose. *The Journal of Biological Chemistry*.

[13] Bradford M. M. (1976). A rapid and sensitive method for the quantitation of microgram quantities of protein utilizing the principle of protein-dye binding. *Analytical Biochemistry*.

[14] Vararattanavech A., Ketterman A. J. (2003). Multiple roles of glutathione binding-site residues of glutathione S-transferase. *Protein and Peptide Letters*.

[15] Deponte M. (2013). Glutathione catalysis and the reaction mechanisms of glutathione-dependent enzymes. *Biochimica et Biophysica Acta*.

[16] Ellman G. L. (1959). Tissue sulfhydryl groups. *Archives of Biochemistry and Biophysics*.

[17] Luna G. L. (1967). *Histopathology Laboratories, Armed Forces Institute of Pathology Washington, D.C*.

[18] Hong J., Stubbins R. E., Smith R. R., Harvey A. E., Núñez N. P. (2009). Differential susceptibility to obesity between male, female and ovariectomized female mice. *Nutrition Journal*.

[19] Corbould A. (2007). Chronic testosterone treatment induces selective insulin resistance in subcutaneous adipocytes of women. *The Journal of Endocrinology*.

[20] O'Rourke R. W. (2009). Inflammation in obesity-related diseases. *Surgery*.

[21] Hernandez I., Delgado J. L., Diaz J. (2000). 17*β*-Estradiol prevents oxidative stress and decreases blood pressure in ovariectomized rats. *American Journal of Physiology-Regulatory Integrative and Comparative Physiology*.

[22] Alonso A., Fernández R., Moreno M., Ordóñez P., Díaz F., González C. (2007). Leptin and its receptor are controlled by 17*β*-estradiol in peripheral tissues of ovariectomized rats. *Experimental Biology and Medicine*.

[23] Pérez-Torres I., El Hafidi M., Zamora-González J., Infante O., Chavira R., Baños G. (2007). Modulation of aortic vascular reactivity by sex hormones in a male rat model of metabolic syndrome. *Life Sciences*.

[24] Meyer M. R., Clegg D. J., Prossnitz E. R., Barton M. (2011). Obesity, insulin resistance and diabetes: sex differences and role of oestrogen receptors. *Acta Physiologica*.

[25] Sophie J., Bernelot M., Gavin R. (2012). Rapid estrogen receptor signaling is essential for the protective effects of estrogen against vascular injury. *Circulation*.

[26] Homma H., Kurachi H., Nishio Y. (2000). Estrogen suppresses transcription of lipoprotein lipase gene. Existence of a unique estrogen response element on the lipoprotein lipase promoter. *The Journal of Biological Chemistry*.

[27] Rogers N. H., Li J. W. P., Strissel K. J., Obin M. S., Greenberg A. S. (2009). Reduced energy expenditure and increased inflammation are early events in the development of ovariectomy-induced obesity. *Endocrinology*.

[28] Ramirez I. (1981). Estradiol-induced changes in lipoprotein lipase, eating, and body weight in rats. *The American Journal of Physiology*.

[29] Fan W., Yanase T., Nomura M. (2005). Androgen receptor null male mice develop late-onset obesity caused by decreased energy expenditure and lipolytic activity but show normal insulin sensitivity with high adiponectin secretion. *Diabetes*.

[30] Eisner J. R., Dumesic D. A., Kemnitz J. W., Colman R. J., Abbott D. H. (2003). Increased adiposity in female rhesus monkeys exposed to androgen excess during early gestation. *Obesity Research*.

[31] Movérare-Skrtic S., Venken K., Andersson N. (2006). Dihydrotestosterone treatment results in obesity and altered lipid metabolism in orchidectomized mice. *Obesity*.

[32] Stubbins R. E., Holcomb V. B., Hong J., Núñez N. P. (2012). Estrogen modulates abdominal adiposity and protects female mice from obesity and impaired glucose tolerance. *European Journal of Nutrition*.

[33] Stubbins R. E., Najjar K., Holcomb V. B., Hong J., Núñez N. P. (2012). Oestrogen alters adipocyte biology and protects female mice from adipocyte inflammation and insulin resistance. *Diabetes, Obesity and Metabolism*.

[34] Gregor M. F., Hotamisligil G. S. (2011). Inflammatory mechanisms in obesity. *Annual Review of Immunology*.

[35] Rocha V. Z., Folco E. J. (2011). Inflammatory concepts of obesity. *International Journal of Inflammation*.

[36] Higdon J. V., Frei B. (2003). Obesity and oxidative stress: a direct link to CVD?. *Arteriosclerosis, Thrombosis, and Vascular Biology*.

[37] Furukawa S., Fujita T., Shimabukuro M. (2004). Increased oxidative stress in obesity and its impact on metabolic syndrome. *Journal of Clinical Investigation*.

[38] Venditti P., Di Stefano L., Di Meo S. (2013). Mitochondrial metabolism of reactive oxygen species. *Mitochondrion*.

[39] Huang T.-T., Yasunami M., Carlson E. J. (1997). Superoxide-mediated cytotoxicity in superoxide dismutase-deficient fetal fibroblasts. *Archives of Biochemistry and Biophysics*.

[40] Borrás C., Gambini J., Gómez-Cabrera M. C. (2005). 17*β*-oestradiol up-regulates longevity-related, antioxidant enzyme expression via the ERK1 and ERK2[MAPK]/NF*κ*B cascade. *Aging Cell*.

[41] Yu J., Zhao Y., Li B., Sun L., Huo H. (2012). 17*β*-Estradiol regulates the expression of antioxidant enzymes in myocardial cells by increasing Nrf2 translocation. *Journal of Biochemical and Molecular Toxicology*.

[42] Kumar P., Kale R. K., Baquer N. Z. (2011). Estradiol modulates membrane-linked ATPases, antioxidant enzymes, membrane fluidity, lipid peroxidation, and lipofuscin in aged rat liver. *Journal of Aging Research*.

[43] Brandes R. P., Mügge A. (1997). Gender differences in the generation of superoxide anions in the rat aorta. *Life Sciences*.

[44] Pajović S. B., Saicić Z. S. (2008). Modulation of antioxidant enzyme activities by sexual steroid hormones. *Physiological Research*.

[45] Pajović S. B., Saičić Z. S., Spasić M. B., Petrović V. M. (2003). The effect of ovarian hormones on antioxidant enzyme activities in the brain of male rats. *Physiological Research*.

[46] Pérez-Torres I., Roque P., El Hafidi M., Diaz-Diaz E., Baños G. (2009). Association of renal damage and oxidative stress in a rat model of metabolic syndrome. Influence of gender. *Free Radical Research*.

[47] Lundholm L., Putnik M., Otsuki M. (2008). Effects of estrogen on gene expression profiles in mouse hypothalamus and white adipose tissue: target genes include glutathione peroxides 3 and cell death-inducing DNA fragmentation factor, *α*-subunit-like effector A. *The Journal of Endocrinology*.

[48] Amengual-Cladera E., Lladó I., Gianotti M., Proenza A. M. (2012). Retroperitoneal white adipose tissue mitochondrial function and adiponectin expression in response to ovariectomy and 17*β*-estradiol replacement. *Steroids*.

[49] Pinto R. E., Bartley W. (1969). The nature of the sex-linked differences in glutathione peroxidase activity and aerobic oxidation of glutathione in male and female rat liver. *The Biochemical Journal*.

[50] Baltgalvis K. A., Greising S. M., Warren G. L., Lowe D. A. (2010). Estrogen regulates estrogen receptors and antioxidant gene expression in mouse skeletal muscle. *PLoS ONE*.

[51] Signorelli S. S., Neri S., Sciacchitano S. (2006). Behaviour of some indicators of oxidative stress in postmenopausal and fertile women. *Maturitas*.

[52] Lubos E., Loscalzo J., Handy D. E. (2011). Glutathione peroxidase-1 in health and disease: from molecular mechanisms to therapeutic opportunities. *Antioxidants and Redox Signaling*.

[53] Lee Y. S., Kim A. Y., Choi J. W. (2008). Dysregulation of adipose glutathione peroxidase 3 in obesity contributes to local and systemic oxidative stress. *Molecular Endocrinology*.

[54] Beutler E., Dunning D., Dabe I. B., Forman L. (1988). Erythrocyte glutathione S-transferase deficiency and hemolytic anemia. *Blood*.

[55] Aniya Y., Imaizumi N. (2011). Mitochondrial glutathione transferases involving a new function for membrane permeability transition pore regulation. *Drug Metabolism Reviews*.

[56] Curtis J. M., Grimsrud P. A., Wright W. S. (2010). Downregulation of adipose glutathione S-tansferase A4 leads to increased protein carbonylation, oxidative stress, and mitochondrial dysfunction. *Diabetes*.

[57] Hayes J. D., Flanagan J. U., Jowsey I. R. (2005). Glutathione transferases. *Annual Review of Pharmacology and Toxicology*.

[58] Chanas S. A., Jiang Q., McMahon M. (2002). Loss of the Nrf2 transcription factor causes a marked reduction in constitutive and inducible expression of the glutathione S-transferase Gsta1, Gsta2, Gstm1, Gstm2, Gstm3 and Gstm4 genes in the livers of male and female mice. *The Biochemical Journal*.

[59] Yin F., Sancheti H., Cadenas E. (2012). Mitochondrial thiols in the regulation of cell death pathways. *Antioxidants and Redox Signaling*.

[60] Olafsdottir K., Reed D. J. (1988). Retention of oxidized glutathione by isolated rat liver mitochondria during hydroperoxide treatment. *Biochimica et Biophysica Acta (BBA)—General Subjects*.

[61] Sies H. (2014). Role of metabolic H_2_O_2_ generation: redox signaling and oxidative stress. *The Journal of Biological Chemistry*.

[62] Wang X., Dykens J. A., Perez E. (2006). Neuroprotective effects of 17*β*-estradiol and nonfeminizing estrogens against H_2_O_2_ toxicity in human neuroblastoma SK-N-SH cells. *Molecular Pharmacology*.

[63] Behl C., Skutella T., Lezoualc'h F. (1997). Neuroprotection against oxidative stress by estrogens: structure-activity relationship. *Molecular Pharmacology*.

[64] Suh J. H., Shenvi S. V., Dixon B. M. (2004). Decline in transcriptional activity of Nrf2 causes age-related loss of glutathione synthesis, which is reversible with lipoic acid. *Proceedings of the National Academy of Sciences of the United States of America*.

[65] Abdul-Rasheed O. F., Al-Shamma G. A., Zillo B. H. (2010). Serum *γ*-glutamyltransferase as oxidative stress marker in pre-and postmenopausal Iraqi women. *Oman Medical Journal*.

[66] Taskiran D., Evren V. (2012). Estradiol protects adipose tissue-derived stem cells against H_2_O_2_-induced toxicity. *Journal of Biochemical and Molecular Toxicology*.

[67] Richardson T. E., Yu A. E., Wen Y., Yang S.-H., Simpkins J. W. (2012). Estrogen prevents oxidative damage to the mitochondria in Friedreich's ataxia skin fibroblasts. *PLoS ONE*.

[68] Sugioka K., Shimosegawa Y., Nakano M. (1987). Estrogens as natural antioxidants of membrane phospholipid peroxidation. *FEBS Letters*.

